# Feasibility, Acceptability, and Preliminary Outcomes of a Mobile Adaptation of a Relational Savoring Intervention to Prevent Loneliness in College Students: Mixed Methods Pilot Study

**DOI:** 10.2196/70528

**Published:** 2025-09-08

**Authors:** Brenda Nguyen, Jocelyn Lai, Hana Qureshi, Christopher Marcotullio, Sina Labbaf, Yuning Wang, Salar Jafarlou, Nikil Dutt, Amir M Rahmani, Jessica L Borelli

**Affiliations:** 1 Department of Psychological Science, School of Social Ecology, University of California, Irvine, 4201 Social and Behavioral Sciences Gateway, Irvine, CA, 92697, United States, 1 203-887-8857; 2 Department of Computer Science, Donald Bren School of Information and Computer Sciences, University of California, Irvine, California, United States; 3 Department of Computing, University of Turku, Turku, Finland; 4 Sue and Bill Gross School of Nursing, University of California, Irvine, California, United States

**Keywords:** relational savoring, loneliness, connectedness, mobile, college students

## Abstract

**Background:**

Rates of loneliness have risen sharply since the onset of the COVID-19 pandemic, largely due to disruptions in social relationships and daily routines, with college students experiencing some of the greatest increases. While prevention programs targeting loneliness have been developed, their success has been limited. One promising approach may lie in enhancing the quality of existing relationships rather than simply increasing social interactions during periods of acute loneliness. Relational savoring, an intervention rooted in attachment theory and positive psychology, aims to deepen feelings of connection by encouraging individuals to reflect on positive interpersonal experiences.

**Objective:**

This study aimed to evaluate the feasibility, acceptability, and preliminary outcomes of a mobile health adaptation of relational savoring, termed mSavorUs (developed by Amir Rahmani), designed to prevent and reduce loneliness among college students.

**Methods:**

A randomized controlled pilot study was conducted with a diverse sample of 29 college students (43.3% Latinx, 40% Asian American, and 16.7% White). The intervention leveraged a smart ring, smartwatch, and smartphone app to enable just-in-time delivery of relational savoring prompts, alongside continuous monitoring of loneliness-related indicators (eg, physiological activity, sleep, and behavior). Aim 1 involved a thematic analysis of participant feedback regarding the utility, benefits, and challenges of both mSavorUs and the monitoring tools. Aim 2 examined the intervention’s effects on loneliness and perceived connectedness.

**Results:**

For aim 1, qualitative findings suggested that participants found the content of mSavorUs (developed by Amir Rahmani) rewarding and helpful; however, the timing of the intervention was often experienced as disruptive. For aim 2, quantitative analyses revealed no significant reductions in loneliness or increases in connectedness, indicating the need for adjustments to the intervention delivery method.

**Conclusions:**

Although participants found the intervention content valuable, the just-in-time delivery format may have limited its effectiveness. Future iterations should consider alternative timing or delivery strategies to maximize program benefits.

## Introduction

### Background

As of 2019, approximately 60% of American adults met the clinical criteria for loneliness [1], marking it as a significant public health concern [2,3]. Loneliness is a subjective experience that may stem from a lack of social contact [4] or from the perception that one’s social relationships are insufficient in quality or quantity [5]. It is associated with a range of negative physical and mental health outcomes, including increased risk of mortality [6,7]. Although research on loneliness has traditionally focused on older adults, recent global data indicate that young adults report higher levels of loneliness than older individuals [8]. Young adulthood is a developmental period marked by both opportunity and vulnerability. Challenges related to identity development, forming new relationships, living independently, managing financial responsibilities, and navigating academic pressures may heighten feelings of loneliness during this stage of life [9]. At the same time, this period presents a valuable window for establishing healthy coping and relational habits. Accordingly, this study aimed to evaluate the effectiveness of a novel, in-the-moment intervention designed to reduce loneliness and enhance connectedness among young adults.

### Existing Loneliness Prevention and Intervention Programs

Interventions aimed at preventing or reducing loneliness have shown some effectiveness among youth and emerging adults [10]. Many existing programs focus on promoting successful social interactions and fostering new social connections [11]. However, despite their intuitive appeal, a meta-analysis found that the overall effect sizes of these interventions on reducing loneliness are small [12]. These findings suggest that simply increasing the quantity of social interactions or perceived social support may not sufficiently address the subjective experience of loneliness. Instead, individuals struggling with loneliness may benefit more from interventions that help them derive deeper meaning from existing relationships, particularly when delivered at moments of heightened need.

Loneliness can persist even in the presence of frequent social interaction if negative social cognitions diminish the perceived quality of relationships. If individuals do not feel safe, secure, or supported by others, they may continue to feel lonely despite being socially engaged. Therefore, interventions that enhance perceptions of relational quality, by fostering feelings of connection, security, and emotional closeness, may represent a more effective approach. However, limited research has explored the efficacy of such strategies for alleviating loneliness, underscoring the need for further investigation in this area.

### Relational Savoring

Grounded in attachment theory, relational savoring is a brief, guided intervention designed to help individuals reflect on positive memories with relational and attachment-relevant content [13,14]. This technique encourages participants to re-experience moments in which they either provided or received safety, security, and support, thereby amplifying the positive emotions and cognitions associated with those experiences [14]. These reflections may center on significant milestones (eg, saying goodbye to parents before leaving for college) or everyday interactions that might otherwise go unnoticed (eg, assisting an older person with loading groceries). The relational savoring protocol follows 5 standardized steps, including sensory focus, emotional focus, meaning-making, future orientation, and mind-wandering, to facilitate deep and intentional engagement with the memory.

Relational savoring has been evaluated in multiple randomized controlled trials across diverse populations, including parents of young children [15], individuals in long-distance relationships [16], adolescents in residential treatment [17], and older adults [18]. The intervention has been delivered both via a website [15,16] and in person [17-19], demonstrating benefits, such as increased positive emotion, enhanced feelings of closeness, greater relationship satisfaction, and reduced physiological reactivity [15-18]. Importantly, relational savoring appears effective even when the other person in the target relationship is not physically present [20]. By guiding individuals to focus on meaningful moments of connection, relational savoring promotes a deeper sense of closeness and increases help-seeking behavior [16]. In doing so, it may help individuals appreciate the strengths of their existing relationships, thereby offering a promising strategy for preventing and alleviating loneliness.

### Increasing Accessibility Through Digital Just-in-Time Interventions

Intervention accessibility is important to young adults; with the use of digital mental health tools on the rise over the past few years [21], several technology-based interventions addressing loneliness among young adults have also been explored by adapting existing interventions into online platforms. These digital interventions have been shown to provide similar benefits to the original interventions, particularly increasing access to low-income individuals and young adults who may prefer digital tools [21,22]. Just-in-time (JIT) interventions that leverage digital tools are designed to carefully time the delivery of interventions to moments when they may be relevant to increase the efficacy of the intervention [23]. Such approaches aim to provide appropriate real-time support in moments of need by tracking changes in individuals’ internal state based on various cues, such as physiological changes [23]. Studies have shown that JIT interventions can have greater efficacy than non-JIT interventions [24,25].

Currently, the use of JIT interventions to alleviate feelings of loneliness and increase feelings of connectedness has not been tested. With 38% of US teenagers and young adults reporting that they already use mobile apps for well-being [26], there is reason to believe that the development of effective mobile-based interventions for loneliness may be feasible, familiar, and cost-effective.

### Overview of Study Aims

Given the growing risks associated with heightened feelings of loneliness and the limited effectiveness of existing interventions, a more accessible and targeted approach focusing on individuals’ subjective perceptions of their relationships may offer a promising path forward. To this end, we developed mSavorUs, a smartphone app designed to deliver relational savoring in a mobile format aimed at preventing and reducing loneliness among emerging adults. Building on prior work developing personalized, predictive models of mental health and well-being [27-30], we integrated a ubiquitous monitoring system that combined ecological momentary assessments (EMAs), passive mobile sensing, and wearable physiological sensors to capture real-time indicators of health and emotional state. The design of this system was predicated on well-established links between sleep disturbances [31], physical activity [32], and physiology [33,34] with loneliness. This system was paired with a proactive mobile intervention that delivered relational savoring exercises JIT—specifically when the monitoring system detected potential episodes of loneliness. We pursued two primary aims:

1. Aim 1: Qualitatively assess the feasibility and acceptability of mSavorUs by evaluating participants’ perceptions of the utility, benefits, and challenges of each feature of the intervention, including the monitoring systems.
2. Aim 2: Quantitatively evaluate the preliminary impact of mSavorUs on loneliness and social connectedness. We hypothesized that participants randomized to the mSavorUs intervention group would report greater reductions in loneliness and greater increases in feelings of social connectedness compared to those in the monitoring-only control group.

## Methods

### Participants

Undergraduate students were recruited from a large West Coast university in the United States. Recruitment was accomplished through the distribution of flyers and posting announcements about the study on class web pages for large undergraduate courses. Participants were eligible if they were between the ages of 18 and 22 years and were fluent in English. Due to the system requirements of the app designed for the study, participants were also required to use an Android smartphone with an operating system of 6.0 or higher. To create a more homogenous sample reflecting emerging adulthood, participants could not be parents, married, or returning to school after a 3-year or longer absence. Individuals who had participated in a similar previous study were also ineligible. We recruited a pilot sample size. A total of 37 students were enrolled in the study. Eight withdrew participation over the course of the study (n=29; mean 19.93, SD 1.22 years; 13 male, 16 female). Of the remaining participants, 43.3% (n=13) of participants identified as Latinx, 43.3% (n=12) as Asian American, and 17.2% (n=5) of participants identified as White.

### Procedure

Participants were enrolled in the study between late January and early February of 2021. Study procedures lasted 22 weeks. First, participants completed an in-person baseline assessment, reporting on baseline levels of loneliness and social connection. They were provided with wearable smart devices (ie, Oura Ring and Samsung Watch) and installed study-relevant phone apps (ie, *m*SavorUs, AWARE [developed by AR], and wearable device apps). Table S1 in Multimedia Appendix 1 provides a brief overview of the apps and devices. Each participant was provided with a study ID that had a preassigned group through a randomization generator. Participants were assigned their IDs based on their enrollment order and were unaware of what group they were placed into.

After enrollment, participants completed a 6-week-long monitoring period, wearing smart devices and completing 5 interval-based EMAs daily. The EMAs consisted of brief questions that assessed emotions, feelings of loneliness, and social connectedness. Participants were asked between 20 and 40 questions in each EMA assessment (eg, morning surveys asked about sleep quality), but this study focuses on just 2 questions asked in each EMA assessment.

The algorithm was developed using objective data collected from smartphones, smartwatches, and smart rings (for algorithm details, see [35]). During the monitoring period, these data were analyzed for associations with self-reported loneliness, as measured via EMAs. Objective features included various sleep metrics, heart rate variability, phone usage patterns, physical location, and types of phone notifications. A binary classification label (lonely vs not lonely) was generated based on median EMA-reported loneliness scores. Personalized random forest models were trained for each participant, using the first 50% of their individual data, while incorporating data from other participants to train the model and the remaining 50% for testing. This hybrid approach captured both individual and group-level patterns, yielding an average model accuracy of 82% (see [35] for further details). During the intervention phase, the algorithm was used to trigger mSavorUs prompts when participants’ objective data indicated potential loneliness risk. Prompts could be delivered between 9 AM and 9 PM, regardless of the participant’s current context (eg, at home, in class, shopping, or resting).

Participants then entered the next phase of the study, which lasted 4 weeks. During this time, the monitoring group (n=14) continued with the same procedures while the relational savoring group (n=15) additionally completed the JIT intervention. Specifically, the mSavorUs group was prompted through the app to complete the relational savoring intervention during periods of suspected loneliness elevations, determined by sensing-based indicators that had previously been associated with their own above-median level of loneliness during the monitoring phase (ie, personalized algorithm). The timing of the JIT intervention was guided by continuous monitoring of each participant’s psychological and physical state using a personalized mental health navigator framework, described above [29,35]. When objective indicators or EMA loneliness reports exceeded a participant-specific threshold usually above the median within a defined time window, the system delivered a JIT prompt to complete the mSavorUs intervention. If the participant did not respond to the JIT prompt by completing the intervention, they would receive a second and a third prompt within that hour-long interval.

**Figure 1.**
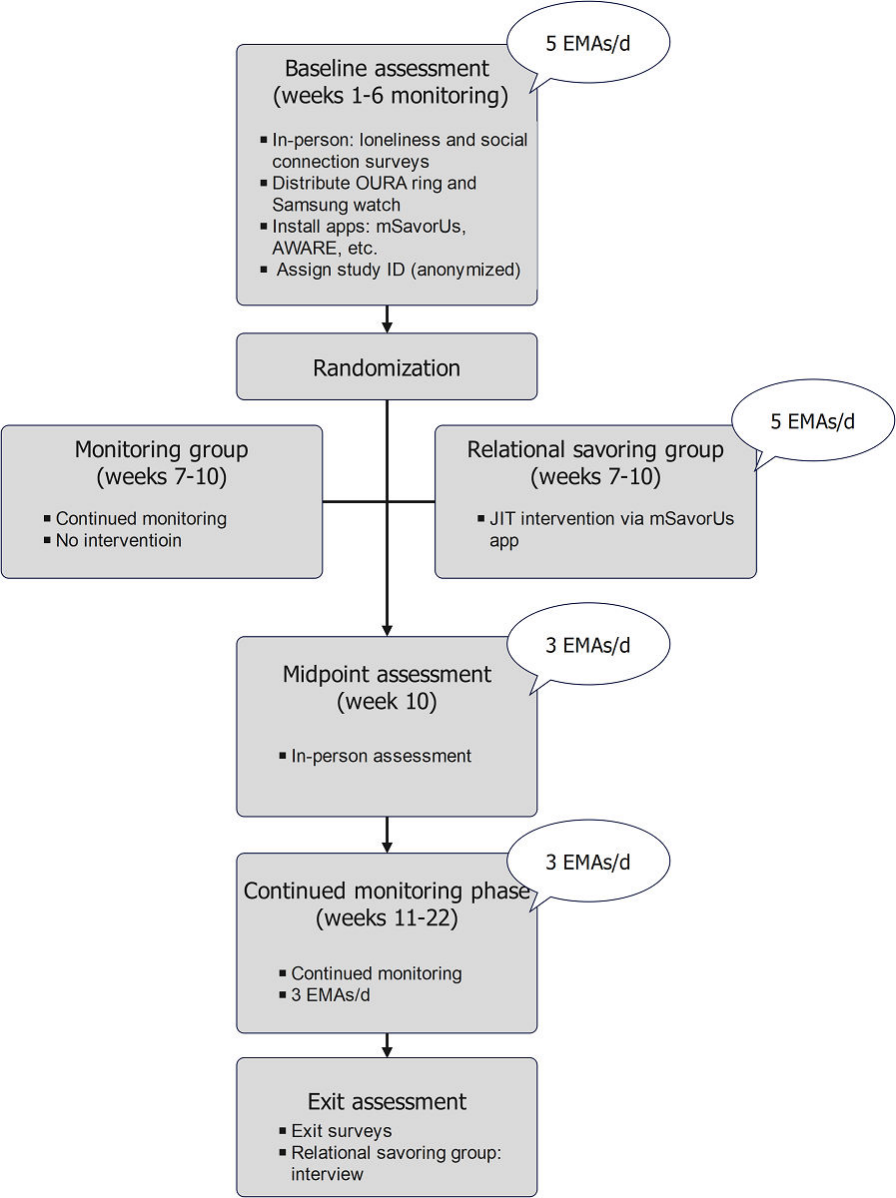
Study Timeline. EMA: ecological momentary assessment; JIT: Just-in-Time.

To reduce participant burden, if a participant’s sensing data indicated the potential for elevated loneliness multiple times within an hour-long interval but they had already completed the intervention, they would not be prompted to complete it again. At the end of the 4-week intervention period, both groups completed a midpoint assessment, like that of the baseline assessment. All participants then completed 12 weeks of continued monitoring. During this phase, no intervention was provided, and the number of daily EMAs was reduced to 3 in order to reduce participation burnout. An exit assessment was completed at the end of the study during an in-person session where participants returned their devices and uninstalled all study-related phone apps. The mSavorUs group completed an interview about their experiences with the intervention. The study timeline is depicted in Figure 1.

### mSavorUs Intervention

Once randomized to the mSavorUs condition, participants met for an individual session with a researcher in which they were guided through an in-person administration of relational savoring, following the protocol established in prior research [13]. Following this in-person session, researchers demonstrated the use of the mSavorUs app. Researchers explained how the app notifications would work and how participants could respond to the notifications by completing the intervention.

Each time they completed the intervention on the mSavorUs app, pursuant to the protocol of previous administrations of relational savoring [13], participants first completed a brief mindfulness breathing exercise. Then they provided written responses to a series of 5 prompts designed to build appreciation for moments of relational connection. The 5 prompts corresponded to the 5 reflection steps used in prior trials of relational savoring [13]. In this study, participants were asked to think of different memories to reflect on each time they used the mSavorUs app. Participants also had the option to upload pictures related to the memories they shared and had access to them at all times while using the app.

### Measures

#### Feasibility and Acceptability of Relational Savoring Intervention.

At the exit assessment (after week 22 of study participation), the mSavorUs group completed brief, semistructured individual interviews in which they answered open-ended questions about their experience. Interviews were audiorecorded, transcribed, and had notes taken during the process. Interviews consisted of 22 questions probing feasibility (eg, What barriers did you encounter while engaging in this activity?) and acceptability (eg, What did you like about this activity? What did you dislike?). Trained researchers conducted the interviews, which focused on questions regarding the utility, benefits, and challenges of each feature of the intervention along with their experience with all the features of the monitoring systems (for complete list of questions, see Table S2 in Multimedia Appendix 1).

### Baseline, Midpoint, Exit Assessment of Loneliness and Social Connection

#### University of California Los Angeles Loneliness Scale

The University of California Los Angeles (UCLA) Loneliness Scale is a widely used 3-item measure of perceived loneliness (“How often do you feel you are left out?” [36]). Items were rated on a 3-point Likert scale (1=hardlyever, 2=some of the time, 3=often) and summed, where higher scores reflect greater loneliness (Cronbach α at baseline=0.69, at midpoint=0.58, at endpoint=0.68).

#### The Social Connectedness Scale

This 8-item scale assesses the degree to which individuals feel connected to others in their social environment “I feel disconnected from the world around me” [37]. Items are rated using a 5-point Likert scale (1=strongly agree, 6=strongly disagree, reverse-scored and summed, where greater scores indicate higher levels of perceived social connectedness (α_1baseline_=0.95, α_2midpoint_=0.97, and α_3endpoint_=0.95).

### Multimodal Assessments of Health, Feelings, and Behavior

#### Physiology, Sleep, and Behavioral Patterns

Participants were fitted with the Oura Ring and Samsung Gear Sport smartwatch and downloaded the corresponding mobile apps in an effort to capture an accurate depiction of their daily physical habits, sleep, and health. The Oura ring was used in this study to assess sleep quality by measuring sleep duration, average heart rate during sleep, and heart rate variability during sleep [38]. The Samsung Gear Sport smartwatch was used in this study to measure daily heart rate and daily heart rate variability using sensors and a pedometer system [39].

#### Mobile Activity

The AWARE app [40,41] passively collected biometric data and logged daily routines on participants’ smartphones. The data collected includes social relationship data (eg, amount of time spent with other people), daily rhythms (eg, routines such as going out vs staying in one’s home), and phone interactions (eg, amount of time spent texting, calling, and app browsing). The AWARE framework has been studied and validated [42]. The connection between the AWARE app and research servers was encrypted to ensure the privacy of participants’ data.

#### Momentary Loneliness and Social Connectedness

Throughout the EMA portion of the study, participants completed assessments of loneliness and perceived social connectedness daily on the mSavorUs app. Loneliness was assessed with a single item, “How lonely do you feel right now?” which was rated on a sliding scale (1-100). Social connectedness was assessed with a single item, “How connected do you feel to others right now?” rated on the same scale.

### Data Analytic Plan

#### Aim One: Feasibility and Accessibility

To address “aim one” and evaluate the feasibility of the JIT mSavorUs intervention, we completed thematic analysis (TA; [43]) applied to the qualitative data. The team of coders (n=6), which comprised postbaccalaureate research staff and undergraduate research assistants, reviewed themes generated from the transcribed interviews. The team followed these steps: (1) identifying unique responses, (2) agreeing upon common themes and subthemes, (3) assigning subthemes to each response, (4) calculating agreement among raters, and (5) reviewing responses.

#### Aim Two: Preliminary Quantitative Outcomes

To evaluate changes in feelings of loneliness and connectedness, we first examined whether self-reported loneliness and connectedness varied across the 3 assessment points (ie, initial monitoring phase [baseline], intervention phase [midpoint], and continued monitoring phase [endpoint]). A repeated measures ANOVA was used to compare changes in scores across assessment points and groups (see Section A in Multimedia Appendix 1 provides information regarding procedures for missing data).

We also examined whether there were changes in momentary loneliness and connectedness across the study period. Multilevel models were conducted in RStudio (Posit, PBC; [44,45]). Our model consisted of 3 levels, with multiple assessments (Level-1) nested within day (Level-2) within persons (Level-3). Group assignment (control group vs mSavorUs group), study phase (initial monitoring, intervention, and continued monitoring), day, and assessment number were included as predictors. An interaction term was included for the group and study phase to test changes over the study phase by group. Day was the total number of days assessed, which enabled us to consider trends over time (154 d in total). The EMA number referred to the assessment for that day, which enabled us to consider trends over the day (5 assessments, later changed to 3, per day). Both survey day and EMA were included as a fixed effect (covariate) to account for change over the assessment as well as how much people’s loneliness and social connectedness may vary within a day.

### Ethical Considerations

The study protocol was approved by the institutional review board at the University of California, Irvine (IRB number 2019‐5153). Participants provided informed consent to participate. All participants were assigned a participant identifier, and all data were stored with that identifier. Identifying data were collected from participants for the purpose of contacting them to schedule visits. Identifiable data were stored on a secure network to which only the research team had access. Participants were compensated according to the following schedule (US $30 for the baseline assessment US $60 for the 4-week observation period, US $120 for the 4-week intervention period, US $30 for the postintervention assessment, US $80 for weeks 1‐4 of postintervention monitoring, US $100 for weeks 5‐8 of postintervention monitoring, US $120 for weeks 9‐12 of postintervention monitoring, and US $30 for the final follow-up assessment). In total, participants could earn up to US $645 for completing all study activities. Participants could also earn up to US $75 in bonuses (US $15 every 4 weeks) for maintaining at least 90% adherence (eg, device use and survey completion) during each of the 5 monitored periods. Compensation was distributed via email.

## Results

### Technological Challenges and Participant Adherence

Throughout the study, several technological challenges impacted data collection and participant engagement. These included temporary device disconnections, battery depletion, and conflicts with mobile app permissions, which occasionally disrupted passive data streams. The most significant technical issue occurred following the initial 4-week monitoring period when a technical error delayed the intervention rollout, necessitating a 2-week extension of the monitoring phase for all participants.

In addition, some participants encountered difficulties submitting their EMAs and intervention responses via the mSavorUs app. These challenges were likely due to factors such as unavailable Wi-Fi during submission, limited data storage capacity in the cloud database, or technical issues associated with the mSavorUs app and related apps (eg, AWARE and the Oura Mobile app).

For instance, on at least 2 occasions, data from AWARE were not being received, requiring the research team to maintain close communication with participants to troubleshoot the problem. In one case, a participant withdrew from the study due to the burden of managing technical difficulties. Other participants experienced issues submitting surveys or intervention responses through mSavorUs, prompting the computer science team to investigate and resolve the issues through direct interaction with affected individuals.

Additional technical challenges included difficulties syncing the Oura ring and situations where participants who obtained new phones during the study needed assistance reconnecting their devices to the apps and integrating data sources. Generally, an increase in technological issues appeared to correspond with greater participant fatigue and burden.

To address these challenges, we implemented a proactive data monitoring strategy via a centralized dashboard. This approach allowed for real-time tracking of data integrity and facilitated prompt issue resolution. A dedicated team of psychology research assistants and computer science technicians collaborated closely, addressing most technical issues as they arose and maintaining participant engagement throughout the study.

### Adherence Rates

The total number of possible observations per individual was 607, comprising 210 prompts during the monitoring period, 140 during the intervention period, and 257 during the continued monitoring period. The overall adherence rate for EMA assessments across the study was 69% (mean 417.96, SD 137.63, range 87‐579 completed out of 602 possible observations). During the initial monitoring phase, adherence was 79% (mean 166.04, SD 35.00, range 60‐202 completed out of 210 possible observations). During the intervention phase, adherence was 75% (mean 105.57, SD 35.97, range 14‐139 completed out of 140 possible observations). During the continued monitoring phase, adherence declined to 57% (mean 146.36, SD 76.09, range 7‐245 completed out of 257 possible observations), indicating some decrease over the extended 12-week period.

### Intervention Completion

Among intervention group participants, adherence to completing the intervention was moderate. Once a JIT prompt was triggered, participants who did not complete the mSavorUs intervention received the prompt up to 3 additional times to maximize completion. During the 4-week intervention period, 1205 notifications were sent, with 219 responses submitted, yielding an 18.17% response rate. The response rate across the 3 JIT prompts was relatively consistent: first prompt (16.02%), second prompt (21.25%), and third prompt (19.59%).

### Aim One: Feasibility and Accessibility

Overall, 2 of 11 participants opted out of being recorded for the postintervention interviews. As a result, 5 research members completed a review of all 385 video transcriptions and notes and compared them. A total of 10 responses only had transcripts with no notes. They confirmed the transcripts and notes matched in content and confirmed the themes and percentage agreements were not affected by either version. As a result, notes were used for the TA procedure while transcripts were mainly used for quoting purposes.

The team identified a total of 223 unique responses and agreed upon common overarching themes and subthemes. Once the themes were identified, all 223 responses were assigned a subtheme. A single response could be assigned to more than one subtheme. The percentage of agreement was computed as the number of matching subthemes present for a single response divided by the number of raters (eg, one response was assigned the subtheme “helpful” by 4/6 raters, yielding a percentage agreement of 67%). In addition, responses with a percent agreement of 50% were discussed to resolve the tie, and ratings were then recalculated. Only data that achieved 80% agreement and higher were included.

Of the 223 responses, 167 (74.89%) unique responses reached a minimum 80% interrater agreement while 56 (25.11%) responses did not and thus were excluded. After factoring in that some responses were coded with multiple themes, a total of 269 responses were included. The TA procedure revealed 3 core themes: benefits, barriers, and improvements. Some of the questions covered during the interviews contained responses that did not fall under any core theme, so they are presented separately (see Section B in Multimedia Appendix 1). A complete list of quotes for each theme and subtheme and their frequencies can be found in Tables S3 and S4 in Multimedia Appendix 1.

#### Benefits

Sample quotes illustrating the core theme of Benefits can be found in Table 1. The subthemes that underlie the overarching theme of benefits included “helpful,” “positive feelings,” “calming, relaxing, meditative,” “focused on the moment,” and “reflect and appreciate good memories.” “Helpful” was a general term participants used to describe a variety of experiences with the mSavorUs intervention (ie, it helped participants feel more positive when they were experiencing stress). Interesting, the majority of the comments that were coded as helpful referred to the introductory session led by researchers where they introduced mSavorUs. “Positive feelings” reflected times when participants felt happier, more joyful, or generally more positive after completing the mSavorUs intervention. “Calming, relaxing, meditative” referred to the state of being elicited by the mSavorUs app—it induced a sense of peacefulness in the participants. Participants’ responses indicated that they enjoyed the mSavorUs intervention because it prompted them to take a moment to step away from daily life and reflect on positive memories, reflected in the code, “focused on the moment.” Finally, “reflect and appreciate good memories” described how participants were able to reflect on positive memories in a more detailed fashion that made them appreciate and enjoy the memory more than before.

**Table 1.** Benefits core theme and subthemes.

Subthemes	Quotes	Frequency (%)
Helpful	*I forgot who I had done it with before, but he helped me understand how I should identify more happy moments and make sure there’s no negative and only has to do with positive and so when I think about those moments even if there’s a little bit of negative I make sure to only focus on the positive*.	13.75
Positive feelings	*It’s like having a tough day, you know. It helps to like kinda force yourself to think more positively, because I know at least in these past 2 weeks it’s been a little bit hectic for me so it’s kinda nice to take a moment to think more positively so I think it was kinda helpful to just [improve] the overall mood*.	3.35
Calming, relaxing,meditative	*What I liked about it was, I liked the mediation aspects. I mean I realised that like, through doing it that I don’t ever really sit down with my thoughts, so it was nice to do that*.	1.86
Focused on the moment	*I liked how it focused on focusing on different senses, and like what emotions you’re feeling. I feel like I don’t usually focus on that stuff in everyday life*.	0.74
Reflect and appreciate good memories	*Oh yeah I think it was because you know I have all these memories in my head, you know. And I can think about them, whenever I want, but I think putting it like, actually writing it down and writing down every single like part of it, and it felt like I was explained the memory to someone else that it was just a lot more fun and it was much more easier to actually like make the memory tangible, I guess, I don’t know if that’s the right word, but it is a lot more, felt more real than just like thinking about [it] in my head like actually getting to like write it down and explain it to someone else made it more interesting*.	3.35

#### Barriers

Quotes illustrating the core theme of “Barriers” can be found in Table 2. A variety of subthemes relating to barriers included “high frequency,” “tech issues,” “lack of memories,” “length of activity,” “time-window,” and “busy.” “High frequency” expressed the participants’ concern with the number of times they were prompted to complete the mSavorUs intervention. “Tech issues” entailed any problems relating to the mSavorUs app (eg, activity submission problems or the app inaccurately stating an activity is available vs unavailable). “Lack of memories” reflected participants having difficulties generating new positive memories to reflect on. “Length of activity” described the activity as taking too much of the participants’ time to complete. Similarly, “time-window” was the concern that participants were unable to complete the activity within the given 1-hour window starting from the first prompt notification they received to complete it. Finally, “busy” represented the participants’ inability to complete the activity due to their daily schedules.

**Table 2.** Barriers core theme and subthemes.

Subthemes	Quotes	Frequency (%)
High frequency	*…when I was getting five or six a day… I was getting a little bit irritated because I like couldn’t think of new memories… But once they reduced it to three like I was feeling better about it*.	5.95
Tech issues	*I tried to do them every single time but there always seemed to be problems, with, like, I would go through and write a super detailed response to each, and then at the end it wouldn’t submit, so like I would go through again, write shorter answers, submit again, and a lot of the time it wouldn’t even send through, and then the ones that did were often like super short responses*.	4.83
Lack of memories	*One thing that I did find was that it was hard for me to keep coming up with positive memories, I don’t know, it was hard for me to brainstorm them after a while. Or they became really vague, like if they happened a long time ago, because I don’t remember specific details. So I think because it was so frequent it was hard to come up with different memories*.	9.29
Length of activity	*Probably just how long it took yeah because you need to be pretty detailed*.	1.49
Time window	*As I mentioned before, um, giving only an hour to complete it. And so I’m getting that notification while I was still asleep, and not even done in the morning check-in um. It made it so I wasn’t able to complete the activity every time I got the notification*.	6.32
Busy	*Sometimes I dislike the timing. I understand that it was supposed to be random but sometimes it came at inconvenient times like I might be texting my friends to go do something and I see it pop up but like I am in the middle of doing something at the same time like a class so it’s a little bit inconvenient at times within that like hour*.	14.13

#### Improvements

Quotes illustrating the core theme of “Improvements” can be found in Table 3. The subthemes relating to improvement were related to the barriers listed above, such as “less tech issues,” “lower frequency,” “wider time window,” “decrease activity length,” and “change activity questions.” “Less tech issues” described the participants’ desire to encounter fewer problems when engaging with the mSavorUs app, such as being able to submit responses without any issues. “Lower frequency” represented the desire to reduce the number of prompt notifications participants received within a single day. “Wider time-window” entailed the participants wanting to have more time to complete the activity than the allotted 1-hour window. On a similar note, “decreased activity length” expressed the desire for the mSavorUs intervention to be shorter and to take up less time to complete (eg, reducing the number of questions asked and requiring fewer details for each response). “Change activity questions” involved participants wanting to modify some of the questions in the mSavorUs intervention to have more inclusive answer choices or for the activity to feel less redundant upon each completion.

**Table 3.** Improvements core theme and subthemes.

Subthemes	Quotes	Frequency (%)
Less tech issues	*I don’t know, I did like the formatting of it, it felt very easy, so that’s good. If it could be, you know, the technical issues worked out*.	4.46
Lower frequency	*I think it was a good feature, I think maybe just like decreasing the frequency*.	8.92
Wider time-window	*Maybe giving a broader um amount of time to complete the activity, because I don’t know I couldn’t do it during my class or stuff like that, because it takes like a moment of like just pure reflection. So giving up like a period to do it within longer than an hour I think, would be helpful*.	7.81
Decrease activity length	*I think shorter activities might have been nice*.	6.32
Change activity questions	*Maybe something like changing out the problem solving. I think probably the reason it felt like a chore was it was the exact same thing over and over and over. Just different name for my memory so maybe like changing out the questions, a little bit like think of a memory of a time you specifically felt this way or think of a time you specifically did this thing or you know, a time you felt excited versus spontaneous so you know different different questions, I think, would have made it more intriguing*.	7.43

### Aim Two: Preliminary Quantitative Outcomes

Table S5 in the Multimedia Appendix 1 includes the means across groups and study phases. For the 2×3 repeated measures ANOVAs, we did not find group differences in reported loneliness over the 3 assessment periods (*F*_2,54_=0.50; *P*=.61; η^2^=0.02), nor were there any differences in reported loneliness across the assessment period overall (*F*_2,54_=0.17; *P*=.84; η^2^=0.006). We also did not find group differences in social connectedness (*F*_2,54_=0.72; *P*=.49; η^2^=0.03), nor were there any differences in social connectedness across the period overall (*F*_2,54_=0.73; *P*=.49; η^2^=0.03).

#### Changes in Momentary Loneliness Over the Assessment Period

Loneliness did not change over time or over the course of the entire assessment period (*β*=−0.02, 95% CI −0.04 to −0.00; *P*=.06), but loneliness did change over the assessments per day (*β*=1.66, 95% CI 1.47-1.85; *P*<.001). Participants reported greater feelings of loneliness by the evening EMA or in EMA surveys sent later in the day.

Groups did not differ in momentary loneliness (*β*=−1.17, 95% CI −9.72 to 7.38; *P*=.79). We did, however, find significant group-by-study phase interaction in momentary loneliness between the initial monitoring period and intervention period (*β*=2.00, 95% CI 0.17-3.83; *P*=.03), and the intervention period and continued monitoring period (*β*=−2.40, 95% CI −4.21 to −0.59; *P*=.009). In probing the simple slopes, for the control group, there were decreases in loneliness during the intervention period compared to the initial monitoring period (*β*=−3.66; *P*<.001). Furthermore, there was an actual increase in loneliness for the control group during the continued monitoring period compared to the intervention period (*β*=1.90; *P*=.02). The control group did not differ in their loneliness at the initial monitoring phase and the continued monitoring phase (*β*=−1.76; *P*=.09).

**Figure 2.**
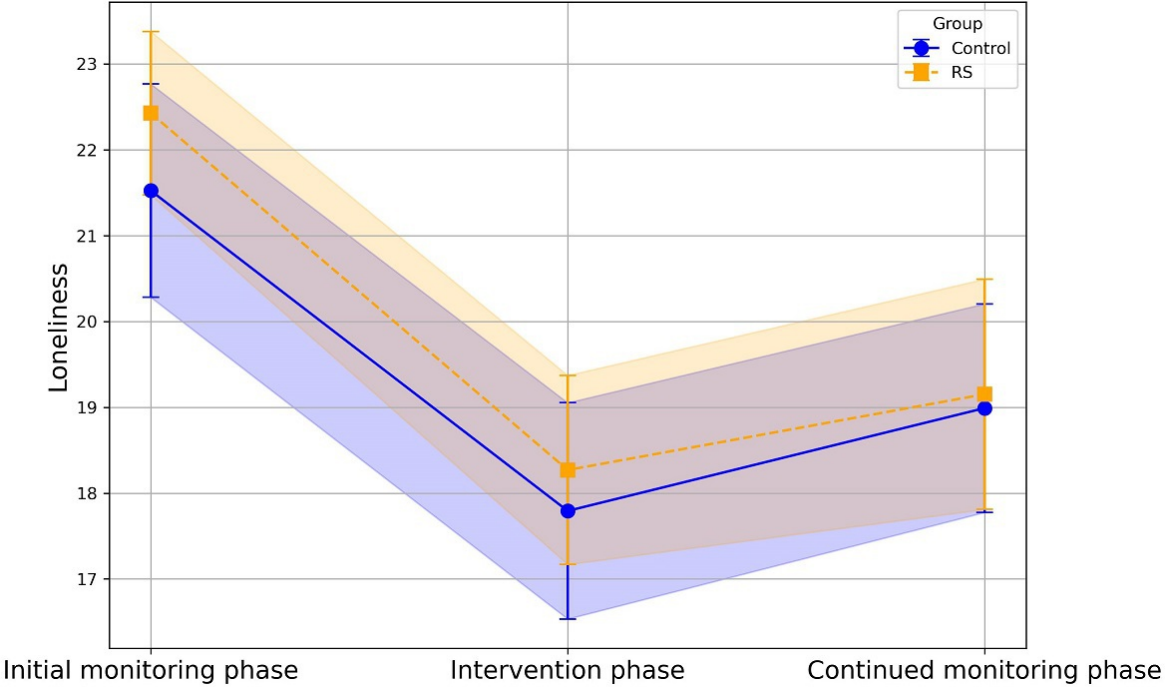
Loneliness across the study period. RS: Relational Savoring.

For the mSavorUs group, they also reported decreases in loneliness during the intervention period compared to the initial monitoring period (*β*=−1.66; *P*=.03) as well as during the continued monitoring phase compared to the initial monitoring period (*β*=−2.16; *P*=.04). There was no difference in loneliness in the intervention period and continued monitoring period for the Relational savoring group (*β*=−0.50; *P*=.55). In other words, the mSavorUs group reported an overall decrease in loneliness from the start of the study (see Tables S6-S9 in Multimedia Appendix 1 for full results). Mean levels of loneliness (Figure 2) for each group are shown across the 3 study phases. Shaded error bands reflect the 95% CIs based on group-wise SEs.

#### Changes in Momentary Connectedness Over the Assessment Period

Connectedness did change over time (*unstandardized β*=0.05, 95% CI 0.02-0.07; *P*=.001) but did not change over the assessments per day (*unstandardized β*=−0.04, 95% CI −0.27 to 0.19; *P*=.74). In other words, participants had a small increase in feelings of connectedness over the study duration, but they did not report differences in feelings of connectedness over the course of the day.

Groups did differ in momentary connectedness (*unstandardized β*=−42.38, 95% CI −53.47 to −31.29; *P*<.001). When testing for interactions with the study phase, we found significant group differences in change across all study phases (the initial monitoring period and intervention period [*unstandardized β*=−4.82, 95% CI −7.17,−2.46; *P* <.001] intervention period and continued monitoring period [*unstandardized β*=−3.87, 95% CI −6.20 to −1.53; *P* <.001] initial monitoring period and continued monitoring period [*unstandardized β*=−8.68, 95% CI −10.77 to −6.60; *P* <.001]). In probing the simple slopes, the control group had increased connectedness during the intervention period compared to the initial monitoring period (*unstandardized β*=4.78; *P*<.001) yet decreased connectedness during the continued monitoring period compared to the intervention period (*unstandardized β*=−3.27; *P*=.003). The control group did not differ in their connectedness at the initial monitoring phase and the continued monitoring phase (*unstandardized β*=1.51; *P*=.27). In summary, the control group reported an increase in connectedness during the intervention period but a decrease in connectedness during the continued monitoring phase.

**Figure 3.**
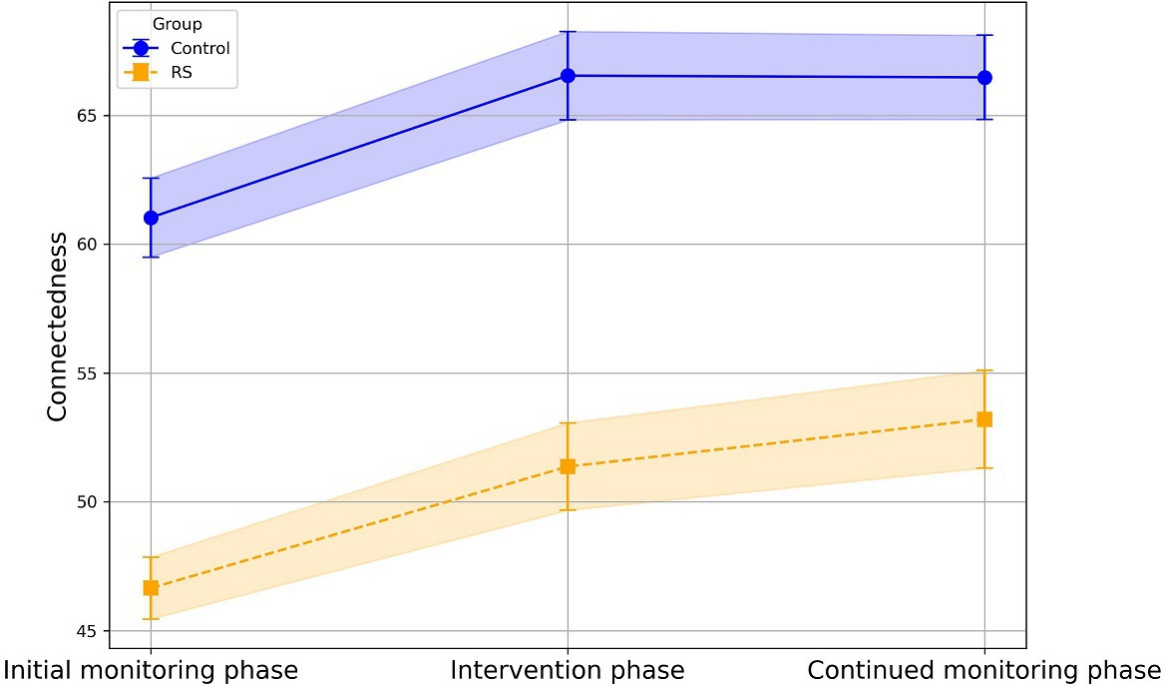
Connectedness across the study period. RS: Relational Savoring.

There was no difference in connectedness comparing the intervention period from the initial monitoring period in the mSavorUs group (*unstandardized β*=−0.04; *P*=.97). Furthermore, contrary to expectations, the mSavorUs group reported a decrease in connectedness during the continued monitoring period compared to the initial monitoring period (*unstandardized β*=−7.18; *P*<.001) as well as a decrease in connectedness during the continued monitoring period compared to the intervention period (*unstandardized β*=−7.14; *P*<.001). Overall, there was a decline in connectedness (see Tables S6-S9 in Multimedia Appendix 1 for full results). Mean levels of connectedness (Figure 3) for each group are shown across the 3 study phases. Shaded error bands reflect the 95% CIs based on group-wise SEs.

## Discussion

### Principal Findings

Loneliness poses significant health risks [6,7], but identifying effective interventions to reduce loneliness has remained challenging [12]. To address this issue, we evaluated the feasibility and efficacy of mSavorUs, a just-in-time tool designed to prevent loneliness and enhance social connectedness. The app leverages relational savoring—an attachment-based intervention aimed at fostering feelings of connectedness within existing relationships [14]. By integrating passive sensing tools, mSavorUs objectively identifies moments of loneliness and delivers the intervention at the most relevant times [35]. Our mixed methods study revealed an interesting pattern of results that are useful in informing future adaptations of mSavorUs. The qualitative findings revealed that while participants enjoyed the substance of the intervention, they found the JIT delivery and technological challenges detracted from the experience. The quantitative findings did not reveal significant impacts of mSavorUs on loneliness or connectedness. In the next 2 sections, we discuss the findings and their implications in greater depth, as well as the limitations of the study.

### Participant Feedback and Challenges

Participants generally viewed the mSavorUs intervention positively. They reported feeling more positive and relaxed during the activity, as it encouraged focusing on the present rather than past or future stressors. However, certain aspects of the JIT format hindered effectiveness. Participants did not always complete the intervention when prompted, often due to busy schedules (eg, academic, work, and extracurricular commitments) or the short time window for response. The long duration of the intervention and the frequency of prompts also posed challenges, particularly during periods of high activity.

Previous studies have similarly noted that a high number of prompts can reduce engagement over time [46]. This finding suggests that while JIT interventions are theoretically promising, they may be less practical when integrated into busy daily lives. Allowing participants to choose when they receive prompts, while accounting for data-driven indicators of loneliness, could increase adherence. For instance, some participants might prefer completing the intervention at bedtime, while others might choose lunchtime. Enhancing user agency in scheduling could improve engagement.

### Quantitative Outcomes: Loneliness and Connectedness

Despite the positive reception, we did not observe significant reductions in loneliness throughout the study. A closer examination revealed that loneliness decreased between the initial monitoring and intervention phases, but unexpectedly, the control group showed a more pronounced reduction compared to the intervention group. Notably, the control group’s loneliness increased during the continued monitoring phase, while the mSavorUs group exhibited a steady decline, suggesting potential benefits from ongoing engagement with the intervention.

Regarding connectedness, significant changes emerged over time. The control group’s connectedness increased between the monitoring and intervention phases but decreased afterward, while the relational savoring group showed a gradual decline across all phases. External factors, such as academic calendar changes, may have influenced these trends, as shifts in routine could affect social dynamics.

Although JIT interventions aim to address individual variability (as seen with health behaviors [47]), reducing loneliness may require broader social initiatives. A meta-analysis reported a linear increase in loneliness among emerging adults over the past 4 decades [48], suggesting that individual interventions, while helpful, may be insufficient on their own.

### Limitations and Future Directions

This study focused on college students, an important population to target given their heightened risk for loneliness [8] and mental health challenges, such as depression [49], particularly during the COVID-19 pandemic. College students’ familiarity with mobile devices made them a suitable group to pilot a mobile-based intervention [21,22]. However, the reliance on a college sample limits generalizability to other groups, especially those less comfortable with technology. Future research should explore the intervention among young adults not in college, as well as middle-aged and older adults who may have lower digital proficiency. In addition, the requirement for an Android device (OS 6.0 or higher) excluded users of other operating systems, such as iPhones and Google phones. Expanding compatibility would improve accessibility and inclusiveness.

Technical challenges, including connectivity issues and app incompatibility, likely contributed to participant fatigue, reducing engagement. Improved integration of app functions, flexible data connection options (Wi-Fi or mobile data), and seamless navigation are priorities for future iterations. Ensuring that the app properly opens and closes and permits consistent connectivity would prevent confusion and increase usability. Moreover, analyzing participants’ preferred engagement times could help optimize prompt delivery, enhancing adherence by aligning intervention prompts with moments of likely availability.

Future studies should also use more reliable metrics to assess loneliness, such as detailed EMA data, given that small sample sizes may compromise the reliability of brief scales like the UCLA Loneliness Scale. Exploring additional analytic techniques could help capture subtle changes in loneliness.

### Conclusion

The mSavorUs intervention shows promise as a mobile tool for reducing loneliness, with potential for sustained benefits even after users discontinue active engagement. Modifying the app’s features, increasing device compatibility, and allowing for more user control over prompt timing could enhance feasibility and user satisfaction. Efforts to modify the intervention may wish to proceed incrementally and with plenty of opportunity for participants to provide feedback. Microtrials could be a useful method for evaluating the short-term impact of mSavorUs app use, while focus groups may provide meaningful participant feedback to inform future improvements. With these improvements, mobile JIT interventions may become a practical and effective method to alleviate loneliness among emerging adults.

## Supplementary material

10.2196/70528 Multimedia Appendix 1 Supporting information regarding the methodology of the study.

## Abbreviations

EMA: ecological momentary assessment
JIT: just-in-time
TA: thematic analysis
UCLA: University of California Los Angeles
