# Supplementary material for: Feasibility, Acceptability, and Preliminary Outcomes of a Mobile Adaptation of a Relational Savoring Intervention to Prevent Loneliness in College Students: Mixed Methods Pilot Study
Source: JMIR Form Res. 2025 Sep 8;9:e70528. doi: 10.2196/70528 (PMC12518887; doi:10.2196/70528)
Supplement: Multimedia Appendix 1 [file formative-v9-e70528-s001.docx]

**Multimedia Appendix 1**

**Descriptive Caption:** *Supporting information regarding the methodology of the study.*

**Table S1. *Outlining Study Materials (Applications & Devices)***

| Oura Ring | The Oura ring collects data on sleep, readiness, and activity. It was used in this study to assess sleep quality by measuring sleep duration, average heart rate during sleep, and heart rate variability during sleep (root mean square of successive differences between normal heartbeats [RMSSD]). |
| --- | --- |
| Samsung Gear Sport Watch | The Samsung Gear Sport smartwatch collects data on sleep and activity by using sensors and a pedometer system. It was used in this study to measure daily heart rate and daily heart rate variability (RMSSD). |
| AWARE App | This app was installed on participants’ phones to passively collect biometric data and log daily routines. The data collected includes movement (steps and exercises), social relationships (amount of time spent with other people based on audio detection and proximity), daily rhythms (routines such as going out vs staying in one’s home), and phone interactions (texting, calling, and app browsing). |
| mSavorUs | This app was installed to prompt participants to complete daily ecological momentary assessments of loneliness, social connectedness, and social isolation. Participants in the intervention condition were also prompted to engage in a guided relational savoring activity when the algorithm detected loneliness. |

**Table S2. *Post-Intervention Interview Questions***

| 1. | How would you describe your experience with the reflection activity? |
| --- | --- |
|  | Could you tell me a little bit why you felt that way? |
| 2. | Do you feel you were properly prepared for this activity from the training session you previously had? |
|  | Explain. Why was this the case? |
|  | If not, what felt new / surprising to you? |
| 3. | What did you like about this activity? What did you dislike about it? |
|  | What ideas/suggestions do you have for improving this feature that you disliked? |
| 4. | What barriers did you encounter while engaging in this activity? |
|  | Issues submitting your responses? |
|  | Difficulties thinking of positive memories? |
|  | Difficulties focusing on the activity or having the time to complete it? |
| 5. | What prevented you from starting the reflection activity when you were prompted to do so? |
| 6. | How did you feel when you received a prompt to complete it? |
| 7. | What were you typically doing when you were prompted to complete the activity? |
| 8. | On average, how often were you prompted to complete this activity daily? |
| 9. | When you did complete the activity, about how long (in minutes) did it take you to complete? |
| 10. | When were you most available to complete this activity? (time of day) |
| 11. | What changes would have allowed or encouraged you to complete the activity more frequently? |
|  | What would have helped you or what would have been helpful for the app to support you in completing the reflection activity? |
| 12. | What additional suggestions do you have for improving this activity if we were to use this again for future studies? |
|  | Shorter activity? |
|  | More interesting/engaging rather than just answering questions? |
|  | Reminders to complete the activity? |
|  | Was the audio recordings helpful in thinking of your positive memories? |
|  | Was it helpful that this activity was in the same app as your daily surveys? |
| 13. | Lastly, would you be open to receiving a brief survey in the future about the overall functionality of the Reflection Activity? |

**Section A. *Data Analytic Plan for Missing Data for Aim 2***

Two participants in the RS group either did not complete the midpoint assessment or did not complete the exit assessment. To address the missing data, we used multiple imputation using 5 imputations. During the multiple imputation process, no clear patterns of missingness were identified. There was also a single missing value for one participant within the SCS.. The mean value was calculated using the participant’s non-missing items for the SCS to complete the missing value. The total SCS score was then calculated per usual.

**Section B. *Additional Information Regarding “No Identified Themes” for Aim 1***

Some interview questions were probed for experiences with the mSavorUs app and its features, resulting in responses that did not fall under any core theme. However, such responses can provide insight to improve overall user experience. On average, participants expressed that it took them between 5-10 minutes to complete the intervention. When asking participants for their preferred time of day to engage in the RS intervention, mixed responses were provided. Some participants said they preferred the early mornings and others said they preferred afternoons, but there was a slight overall preference for evenings before participants went to bed. In regards to the features in the mSavorUs app, most participants agree that reminder notifications to complete the RS intervention were helpful, but to be mindful to not send too many notifications since they also get notifications for other activities on the app. Another feature on the app is an audio recording where participants could play a voice to read them the instructions while engaging in the RS intervention. The majority of the participants stated that they either rarely used or didn’t use the recordings throughout their participation. However, something positive that almost all of the participants agreed upon is that they found it helpful to have the RS intervention in the mSavorUs app with their other participation activities as it helped to stay organized.

**Table S3. *Core Themes and Sub-Themes from Thematic Analysis***

| **Core Themes** | **Sub-Themes** | **Quotes** |
| --- | --- | --- |
| **Benefits** | Helpful | *I forgot who I had done it with before, but he helped me understand how I should identify more happy moments and make sure there's no negative and only has to do with positive and so when I think about those moments even if there's a little bit of negative I make sure to only focus on the positive.* |
|  | Positive Feelings | *It's like having a tough day, you know. It helps to like kinda to force yourself to think more positively, because I know at least in these past 2 weeks its been a little but hectic for me so it's kinda nice to take a moment to think more positively so I think it was kinda helpful to just [improve] the overall mood or day.* |
|  | Calming / Relaxing / Meditative | *What I liked about it was, I liked the mediation aspects. I mean I realized that like, through doing it that I don’t ever really sit down with my thoughts, so it was nice to do that.* |
|  | Focused on the moment | *I liked how it focused on focusing on different senses, and like what emotions you're feeling. I feel like I don’t usually focus on that stuff in everyday life.* |
|  | Reflect & appreciate good memories | *Oh yeah I think it was because you know I have all these memories in my head, you know And I can think about them, whatever I want, but I think putting it like actually writing it down and writing down every single like part of it, and it felt like I was explained the memory to someone else that it was just a lot more fun and it was much more easier to actually like make the memory tangible, I guess, I don't know if that's the right word, but it is a lot more felt more real than just like thinking about in my head like actually getting to like write it down and explain it to someone else made it more interesting.* |
| **Barriers** | High frequency | *…when I was getting five or six a day… I was getting a little bit irritated because I like couldn't think of new memories… But once they reduced it to three like I was feeling better about it.* |
|  | Tech issues | *I tried to do them every single time but there always seemed to be problems, with, like, I would go through and write a super detailed response to each, and then at the end it wouldn't submit, so like I would go through again, write shorter answers, submit again, and a lot of the time it wouldn’t even send through, and then the ones that did were often like super short responses.* |
|  | Lack of memories | *One thing that I did find was that it was hard for me to keep coming up with positive memories, I don’t know, it was hard for me to brainstorm them after a while. Or they became really vague, like if they happened a long time ago, because I don’t remember specific details. So I think because it was so frequent it was hard to coming up with different memories.* |
|  | Length of activity | *Probably just how long it took yeah because you need to be pretty detailed.* |
|  | Time-window | *as I mentioned before, um, giving only an hour to complete it. And so i'm getting that notification while I was still asleep, and not even done in the morning check in um. It made it so I wasn't able to complete the activity every time I got the notification.* |
|  | Busy | *uh sometimes I dislike the timing. I understand that it was supposed to be random but sometimes it came at inconvenient times like I might be texting my friends to go do something and I see it pop up but like I am in the middle of doing something at the same time like a class so it's a little bit inconvenient at times within that like hour.* |
| **Improve-ments** | Less tech issues | *I don’t know, I did like the formatting of it, it felt very easy, so that’s good. If it could be, you know, the technical issues worked out.* |
|  | Lower frequency | *I think it was a good feature, I think maybe just like decreasing the frequency.* |
|  | Wider time-window | *Maybe giving a broader um amount of time to complete the activity, because I don't know I couldn't do it during my class or stuff like that, because it takes like a moment of like just pure reflection. So giving up like a period to do it within longer than an hour I think, would be helpful.* |
|  | Decrease activity length | *I think shorter activities might have been nice.* |
|  | Change activity questions | *maybe something like changing out the problem solving I think probably the reason it felt like a chore was it was the exact same thing over and over and over Just different name for my memory so maybe like changing out the questions, a little bit like think of a memory of a time you specifically felt this way or think of a time you specifically did this thing or you know, a time you felt excited versus spontaneous so you know different different questions, I think, would have made it more intriguing.* |

**Table S4. *Thematic Analysis Code Frequencies***

| **Theme/Code** | **Total** | **% of Total** |
| --- | --- | --- |
| **Benefits** | **62** | **23.05%** |
| Helpful | 37 | 13.75% |
| Positive Feelings | 9 | 3.35% |
| Calming / Relaxing / Meditative | 5 | 1.86% |
| Focused on the moment | 2 | 0.74% |
| Reflect & appreciate good memories | 9 | 3.35% |
| **Barriers** | **113** | **42.01%** |
| High frequency | 16 | 5.95% |
| Tech issues | 13 | 4.83% |
| Lack of memories | 25 | 9.29% |
| Length of activity | 4 | 1.49% |
| Time-window | 17 | 6.32% |
| Busy | 38 | 14.13% |
| **Improvements** | **94** | **34.94%** |
| Less tech issues | 12 | 4.46% |
| Lower frequency | 24 | 8.92% |
| Wider time-window | 21 | 7.81% |
| Decrease activity length | 17 | 6.32% |
| Change activity questions | 20 | 7.43% |
| **Grand Total** | **269** | **100.00%** |

**Table S5. *Descriptives of Loneliness, Support, Connectedness by Group and Study Phase***

|  | **Initial Monitoring Phase**  **(*M*/SD)** | **Intervention Phase**  **(*M*/SD)** | **Continued Monitoring Phase**  **(*M*/SD)** |
| --- | --- | --- | --- |
| **UCLA Loneliness** | Control: 5:00 (1.66)  RS: 5.27 (1.53) | Control: 5.00 (1.41)  RS: 4.97 (1.37) | Control: 4.86 (1.35)  RS: 5.44 (1.64) |
| **Social Connectedness** | Control: 34.43 (13.10)  RS: 34.00 (11.34) | Control: 29.43 (14.27)  RS: 33.45 (11.85) | Control: 36.36 (11.37)  RS: 33.31 (10.62) |
| **Momentary Loneliness** | Control: 21.28 (22.09)  RS: 18.37 (21.26) | Control: 17.70 (19.38)  RS: 16.42 (19.36) | Control: 19.01 (17.09)  RS: 14.33 (19.04) |
| **Momentary Connectedness** | Control: 62.02 (27.45)  RS: 26.14 (24.04) | Control: 67.48 (26.55)  RS: 27.68 (26.17) | Control: 65.88 (25.24)  RS: 21.98 (23.75) |

**Table S6. *Multilevel Model 1 Results of Group and Study Phase Predicting Loneliness***

| ***Predictors*** | ***Estimates*** | ***CI*** | ***p*** |
| --- | --- | --- | --- |
| (Intercept) | 16.71 | 10.65 – 22.76 | **<0.001** |
| Group | -3.17 | -11.68 – 5.33 | 0.465 |
| StudyPhase12 | -3.66 | -5.13 – -2.19 | **<0.001** |
| StudyPhase13 | -1.76 | -3.84 – 0.32 | 0.097 |
| SurveyDay | -0.02 | -0.04 – 0.00 | 0.064 |
| EMA | 1.66 | 1.48 – 1.84 | **<0.001** |
| Group × StudyPhase12 | 2.00 | 0.17 – 3.83 | **0.032** |
| Group × StudyPhase13 | -0.40 | -2.02 – 1.21 | 0.626 |
| **Random Effects** | | | |
| σ^2^ | 220.92 | | |
| τ_00_ _Subj_Day_ | 39.43 | | |
| τ_00_ _ID_ | 129.38 | | |
| ICC | 0.43 | | |
| N _Subj_Day_ | 3627 | | |
| N _ID_ | 28 | | |

*Note.* Observations n = 11693; marginal R^2^ = 0.029; conditional R^2^ = 0.450; control group is the reference group; the initial monitoring period is the reference for the Study Phase.

**Table S7. *Multilevel Model 2 Results of Group and Study Phase Predicting Loneliness***

| ***Predictors*** | ***Estimates*** | ***CI*** | ***p*** |
| --- | --- | --- | --- |
| (Intercept) | 13.05 | 6.87 – 19.22 | **<0.001** |
| Group | -1.17 | -9.72 – 7.38 | 0.788 |
| StudyPhase11 | 3.66 | 2.19 – 5.13 | **<0.001** |
| StudyPhase13 | 1.90 | 0.26 – 3.55 | **0.024** |
| SurveyDay | -0.02 | -0.04 – 0.00 | 0.064 |
| EMA | 1.66 | 1.48 – 1.84 | **<0.001** |
| Group × StudyPhase21 | -2.00 | -3.83 – -0.17 | **0.032** |
| Group × StudyPhase23 | -2.40 | -4.21 – -0.59 | **0.009** |
| **Random Effects** | | | |
| σ^2^ | 220.92 | | |
| τ_00_ _Subj_Day_ | 39.43 | | |
| τ_00_ _ID_ | 129.38 | | |
| ICC | 0.43 | | |
| N _Subj_Day_ | 3627 | | |
| N _ID_ | 28 | | |

*Note.* Observations n = 11693; marginal R^2^ = 0.029; conditional R^2^ = 0.450; control group is the reference group; the intervention period is the reference for the Study Phase.

**Table S8. *Multilevel Model 1 Results of Group and Study Phase Predicting Connectedness***

| ***Predictors*** | ***Estimates*** | ***CI*** | ***p*** |
| --- | --- | --- | --- |
| (Intercept) | 62.28 | 54.43 – 70.13 | **<0.001** |
| Group | -37.56 | -48.59 – -26.53 | **<0.001** |
| StudyPhase12 | 4.78 | 2.88 – 6.67 | **<0.001** |
| StudyPhase13 | 1.51 | -1.18 – 4.19 | 0.271 |
| SurveyDay | 0.05 | 0.02 – 0.07 | **0.001** |
| EMA | -0.04 | -0.27 – 0.19 | 0.740 |
| Group × StudyPhase12 | -4.82 | -7.17 – -2.46 | **<0.001** |
| Group × StudyPhase13 | -8.68 | -10.77 – -6.60 | **<0.001** |
| **Random Effects** | | | |
| σ^2^ | 365.69 | | |
| τ_00_ _Subj_Day_ | 65.15 | | |
| τ_00_ _ID_ | 217.66 | | |
| ICC | 0.44 | | |
| N _Subj_Day_ | 3621 | | |
| N _ID_ | 28 | | |

*Note.* Observations n = 11594; marginal R^2^ = 0.407; conditional R^2^ = 0.665; control group is the reference group; the initial monitoring period is the reference for the Study Phase.

**Table S9. *Multilevel Model Results of Group and Study Phase Predicting Connectedness***

| ***Predictors*** | ***Estimates*** | ***CI*** | ***p*** |
| --- | --- | --- | --- |
| (Intercept) | 67.06 | 59.05 – 75.06 | **<0.001** |
| Group12 | -42.38 | -53.47 – -31.29 | **<0.001** |
| StudyPhase11 | -4.78 | -6.67 – -2.88 | **<0.001** |
| StudyPhase13 | -3.27 | -5.39 – -1.15 | **0.003** |
| SurveyDay | 0.05 | 0.02 – 0.07 | **0.001** |
| EMA | -0.04 | -0.27 – 0.19 | 0.740 |
| Group12 × StudyPhase11 | 4.82 | 2.46 – 7.17 | **<0.001** |
| Group12 × StudyPhase13 | -3.87 | -6.20 – -1.53 | **0.001** |
| **Random Effects** | | | |
| σ^2^ | 365.69 | | |
| τ_00_ _Subj_Day_ | 65.15 | | |
| τ_00_ _ID_ | 217.66 | | |
| ICC | 0.44 | | |
| N _Subj_Day_ | 3621 | | |
| N _ID_ | 28 | | |

*Note.* Observations n = 11594; marginal R^2^ = 0.407; conditional R^2^ = 0.665; control group is the reference group; the intervention period is the reference for the Study Phase.
